# Lumpy Skin Disease—An Emerging Cattle Disease in Europe and Asia

**DOI:** 10.3390/vaccines11030578

**Published:** 2023-03-02

**Authors:** Leah Whittle, Rosamund Chapman, Anna-Lise Williamson

**Affiliations:** Institute of Infectious Disease and Molecular Medicine and Division of Medical Virology, Department of Pathology, University of Cape Town, Cape Town 7700, South Africa

**Keywords:** lumpy skin disease virus, poxvirus, vaccine, recombination

## Abstract

Lumpy skin disease virus (LSDV) is a member of the Capripoxvirus genus, mainly infecting cattle and buffalo, which until relatively recently was only endemic in parts of Africa and then spread to the Middle East and lately Europe and Asia. Lumpy skin disease (LSD) is a notifiable disease with a serious impact on the beef industry as it causes mortality of up to 10% and has impacts on milk and meat production, as well as fertility. The close serological relationship between LSDV, goat poxvirus (GTPV) and sheep poxvirus (SPPV) has led to live attenuated GTPV and SPPV vaccines being used to protect against LSD in some countries. There is evidence that the SPPV vaccine does not protect from LSD as well as the GTPV and LSDV vaccines. One of the LSD vaccines used in Eastern Europe was found to be a combination of different Capripoxviruses, and a series of recombination events in the manufacturing process resulted in cattle being vaccinated with a range of recombinant LSDVs resulting in virulent LSDV which spread throughout Asia. It is likely that LSD will become endemic throughout Asia as it will be very challenging to control the spread of the virus without widespread vaccination.

## 1. Introduction

### 1.1. Background

**Figure 1:**
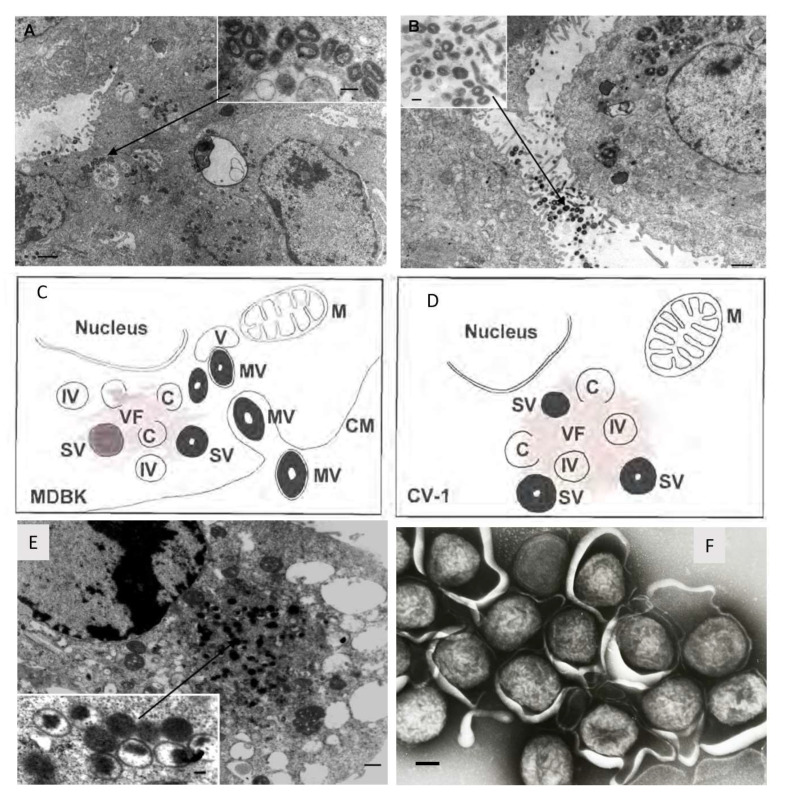
Morphogenesis of recombinant LSDV-Rabies which is identical to LSDV. Permissive bovine Madin-Darby bovine kidney (MDBK) cells were infected with rLSDV-Rabies (1 f.f.u. per cell, 48 h; scale bar = 1 mm) with mature virions inside (**A**) and outside (**B**) the cell. The inserts show high-power virion structure (scale bar = 250 nm). A diagrammatic representation of rLSDV-RG replication in permissive (**C**) non-permissive (**D**) cells. M indicates mitochondria, C indicates crescent shaped membrane which precedes immature virion (IV) formation, (V) indicates vacuoles, (VF) indicates viral factory and electron dense areas where replication and maturation occurs, (IV) indicates immature virion, (MV) indicates mature virion, (CM) indicates cell membrane, and (SV) indicates semi-mature virion. (**E**) Non-permissive primate CV-1 cells were infected with rLSDV-Rabies (1 f.f.u. per cell, 48 h; scale bar = 0.5 mm). The insert is a higher magnification of the ‘viral factory’ (scale bar = 100 nm). Taken from [6,9]. (**F**) LSDV negatively stained(scale bar = 100 nm). Taken by Linda Stannard.

Lumpy skin disease virus (LSDV), which causes lumpy skin disease (LSD) in cattle, belongs to the Capripoxvirus genus of the *Poxviridae* family. Other members of the Capripoxvirus genus are sheeppox virus (SPPV) and goatpox virus (GTPV). Capripoxvirus particles are enveloped and brick-shaped measuring 294 ± 20 nm in length and 262 ± 22 nm in width [1]. Capripoxviruses have a large covalently linked double stranded DNA genome of 150 kb for SPPV and GTPV [2] and 151 kb for LSDV [3]. As seen with other poxviruses, LSDV replicates in the cytoplasm. The viral factory is established and then crescents are formed which will develop into immature virus and then intracellular mature virus. In the Golgi body or early endosome, the intracellular mature viruses are enveloped and then can be exported out of the cell to yield extracellular enveloped viruses [4,5,6]. In non-permissive cells, the life cycle is halted before maturity and although immature virus can be observed, no mature virus develops (refer to Figure 1) [6].

LSD is listed as a notifiable disease by the World Organization for Animal Health (OIE) due to the severe economic impact on the cattle industry. This is due to a number of factors including decreased milk and meat production, abortions, fertility problems, damaged hides and in some cases, death of the animals. LSD can also result in secondary bacterial infections. The resulting trade restrictions and response to outbreaks including vaccination and treatment further amplify the economic losses [7,8].

**Figure 2:**
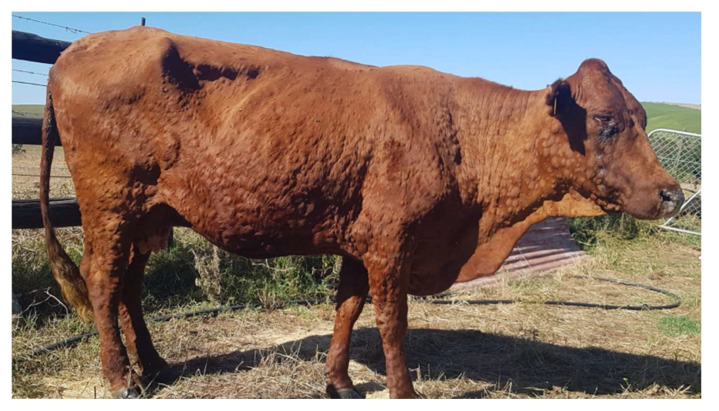
Bonsmara cow with lumpy skin disease—photograph provided by P. B. Kloppers.

LSD manifests in cattle as fever, nasal and ocular discharge, and painful nodular lesions which form on the skin, muscles and mucosal tissue (Figure 2) [10,11]. Light and ultrastructural microscopy studies of infected cattle showed vasculitis and thrombosis to be central in the pathogenesis with replicating viral particles seen in the endothelial cells [5]. 

### 1.2. Host-Range

LSDV is host restricted; its natural hosts are cattle, buffalo and water buffalo [12,13,14]. Antibodies have been detected in black and blue wildebeest, eland, giraffe, greater kudu, African buffalo and other animal species [15]. Giraffe and impala can be experimentally infected and subsequently die from LSD [16]. Recently, LSDV was isolated from nodular lesions of a giraffe which died in a zoo in Vietnam [17]. The authors suggest that it was due to natural infection of the virus, although how this occurred is unknown. The role of wild animals in the epidemiology of LSDV is still not certain.

The host range is slightly broader in cell culture of LSDV. A number of primary cell types derived from lamb and bovine tissue enable the growth of LSDV to high titres and are commonly used for vaccine stock preparations [18,19]. LSDV also grows well in chick chorioallantoic membranes (CAMs) of embryonated eggs, although purification of the virus may need to be extensive which can cause a loss in yields [20]. Various cell lines have been investigated as more convenient alternatives. High titres of LSDV can be obtained when grown in Madin-Darby bovine kidney (MDBK) cells [21]. Unfortunately, this cell line is often contaminated with bovine viral diarrhoea virus (BVDV), and therefore cannot be used for the preparation of vaccine stocks. Removal of BVDV from LSDV cultured in MDBKs can however be achieved after two passages in CAMs [22]. Another is the baby hamster kidney 21 (BHK-21) cell line which was first investigated for LSDV culture in the 1950s–1960s, although it is not frequently used for this purpose [23]. Our laboratory very recently demonstrated that BHK-21 cells can be used for the construction of LSDV recombinants [24]. A recently developed embryonic skin of sheep (ESH-L) cell line was also shown to yield high titres of LSDV [19]. LSDV also replicates in Vero (African green monkey kidney) cells and the ovine testis (OA3.Ts) cell line [18,25]. 

### 1.3. Distribution of LSD

**Figure 3:**
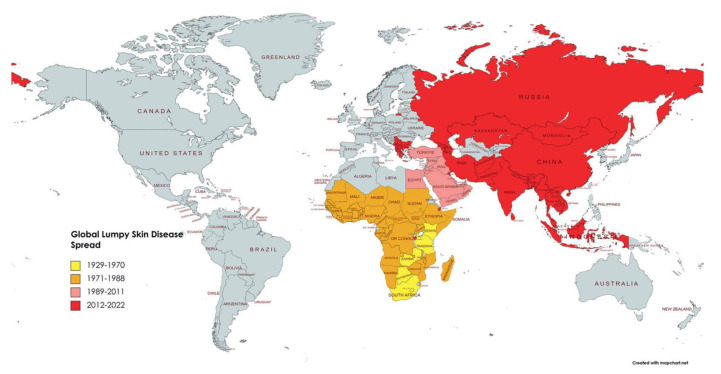
Lumpy skin disease prevalence worldwide and over time from 1929 to 2022. The impacted nations are shown in yellow between 1929 and 1970, orange between 1971 and 1988, pink between 1989 and 2011, and red between 2012 and 2022, with permission from A. Khalafalla, [37].

Last century, LSD was regarded as a disease endemic to Africa with the first report from Northern Rhodesia (now Zambia) in 1929. In 1989, LSD spread to Israel [26] and then further to other countries in the Middle East [27]. In 2015, LSDV was found in the Balkans and in 2016, in Serbia [28]. In the last decade, LSDV spread to countries in Europe, Bangladesh, China, India and Russia [29,30,31,32,33]. Outbreaks of the disease also occurred more recently in Indonesia and Pakistan last year [34] (refer to Figure 3). Outbreaks in parts of Europe have been controlled [35], but it is likely that LSD will become endemic in most parts of Africa and Asia. The outbreak in the Balkans was controlled by vaccination with a live attenuated LSD vaccine based on the Neethling strain. This vaccine provided protection from infection within 14 days of vaccination [35]. However, there is always the concern of further spread of the disease. Vaccination is the best way to control the spread of LSD as subclinical infections are common and identification and removal of infected animals is not always possible [36]. 

### 1.4. Transmission

The main route of LSDV transmission is via a variety of arthropod vectors: ticks, mosquitoes and biting flies have all been implicated in transmission [38,39,40,41,42,43,44]. Climate change may impact on the spread of the arthropod vectors and as a result, the spread of LSDV [45]. To reduce the risk of insect transmission into areas with no LSDV, disinsectisation of vehicles transporting live animals is important [45]. 

LSDV is very stable in the environment, persisting for an extended time in necrotic lesions, desiccated crusts and airdried hides [46]. Direct contact between animals was initially deemed as inefficient for LSDV spread [47]. However, more recent work demonstrated the transmission of a virulent recombinant strain of LSDV (Saratov/2017) between infected bulls and in contact cattle in the absence of a vector [48]. There are also gaps in knowledge on how LSDV can persist in the environment, as there is a report in Russia where two almost identical isolates were identified with a two-year gap, with the implication that the virus circulated in cattle in the region or survived in fomites [49]. The presence of LSDV has been reported in semen after virulent challenge experiments [50] and insemination of heifers with infected semen can cause transmission of LSDV [51]. Of note, vaccination prevented LSDV being shed in semen after virulent challenge [50]. In natural outbreaks, LSDV can be transmitted to bovine foetuses and can result in abortion [52].

### 1.5. Current LSDV Vaccines Used in the Field

LSD is currently controlled using commercial vaccines which are mainly live attenuated LSDVs [53] which originated from the Neethling field strain of LSDV. This virus was attenuated by serial passage in primary lamb kidney cells and CAMs, carried out at the Onderstepoort Veterinary Research Institute (South Africa) in the 1950s–1960s [23,54]. This is known as the Neethling vaccine strain of LSDV (nLSDV). In South Africa, Neethling-like LSDV vaccine strains are marketed as Lumpyvax (MSD Animal Health), Herbivac LS (Deltamune) and Lumpy Skin Disease Vaccine (Onderstepoort Biological Products—OBP) and share 99.5% DNA sequence homology with each other [55]. nLSDV is also the most commonly used LSDV vaccine strain in Europe [56]. 

Due to the cross-reactivity between members of the Capripoxvirus genus, live attenuated GTPV and SPPV are also used to vaccinate cattle against LSDV [57]. GTPV-based vaccines have been reported to show the same protection against LSD as LSDV-based vaccines [58,59]. However, this is not the case with SPPV vaccines. In an experiment where groups of sheep, goats and cattle, vaccinated with Romania SPPV vaccine or nLSDV vaccine, were challenged with corresponding virulent strains, goats or sheep receiving the Romania SPPV vaccine were fully protected against challenge with virulent SPPV and GTPV strains, respectively. However, those cattle that were vaccinated with Romania SPPV vaccine showed only partial protection against LSDV challenge compared to full protection in cattle that received the nLSDV vaccine [60]. These results indicate that either LSDV or GTPV based vaccines could be used to protect from LSD in cattle. 

### 1.6. Efficacy and Safety of Capripoxvirus Vaccines 

A trial was conducted to test five LSDV-based live attenuated vaccines, namely: (1) Lumpy Skin Disease Vaccine [61] (Onderstepoort Biological Products OBP; South-Africa; batch 442); (2) Lumpyvax (MSD-Animal Health; South-Africa; batch BNDM07); (3) Kenyavac (Jordan Bioindustries Center Jovac; Jordan; batch 220115-04); (4) Herbivac LS (Deltamune; South-Africa); (5) Vaccin LSD Neethling O vivant (MCI Santé Animale; Morocco, batch 17BLSDN001). Animals were vaccinated and then challenged with virulent LSDV, 21 days after the last vaccination, and were all protected from challenge. They found that vaccination often resulted in a fever, which varied between the groups. Lumpyvax had a greater response whereas MCI- and Herbivac-vaccinated animals had less response. However, despite this, a Neethling response was seen in 43% of the animals vaccinated with Herbivac and 28% of the animals vaccinated with the MCI vaccine [53]. A Neethling response is the presence of superficial and smaller skin lesions which are distinct from those induced by a virulent field strain that disappear within 2–3 weeks without converting into necrotic scabs or ulcers [62]. Although some regard a Neethling response as negligible [62], these mild reactions have resulted in some hesitancy to use the live vaccines.

Adverse reactions in cattle vaccinated with a SPPV and an unverified LSDV vaccine were reported by farmers in Jordan; however, there is suspicion the LSDV vaccine was a smuggled virulent strain [63]. A randomised controlled field study on over 4000 cattle in Israel confirmed the efficacy of nLSDV vaccines compared to SPPV (x10RM65)-based vaccines. The relative risk of getting LSD in x10RM65 vs. nLSDV vaccinated animals was 2.635 (CI 95% = 1.44–4.82) and 11.2 (2.3–54.7) for severe morbidity. The relative risk of disease in x10RM65 vs. nLSDV vaccinated animals for laboratory confirmed cases was even higher at 4.28 (1.59–11.53), leading to the conclusion that nLSDV is significantly more effective at preventing LSD than x10RM65 [64].

nLSDV and a Kenyan sheep and goat pox virus (KSGP), produced in Ethiopia for the purpose of LSDV vaccination, both failed to protect cattle against LSDV in a challenge study [58]. However, the quality of the nLSDV vaccine batch used was thought to be the cause of the failure [65]. 

### 1.7. Recombinant LSDV in the Field

**Figure 4:**
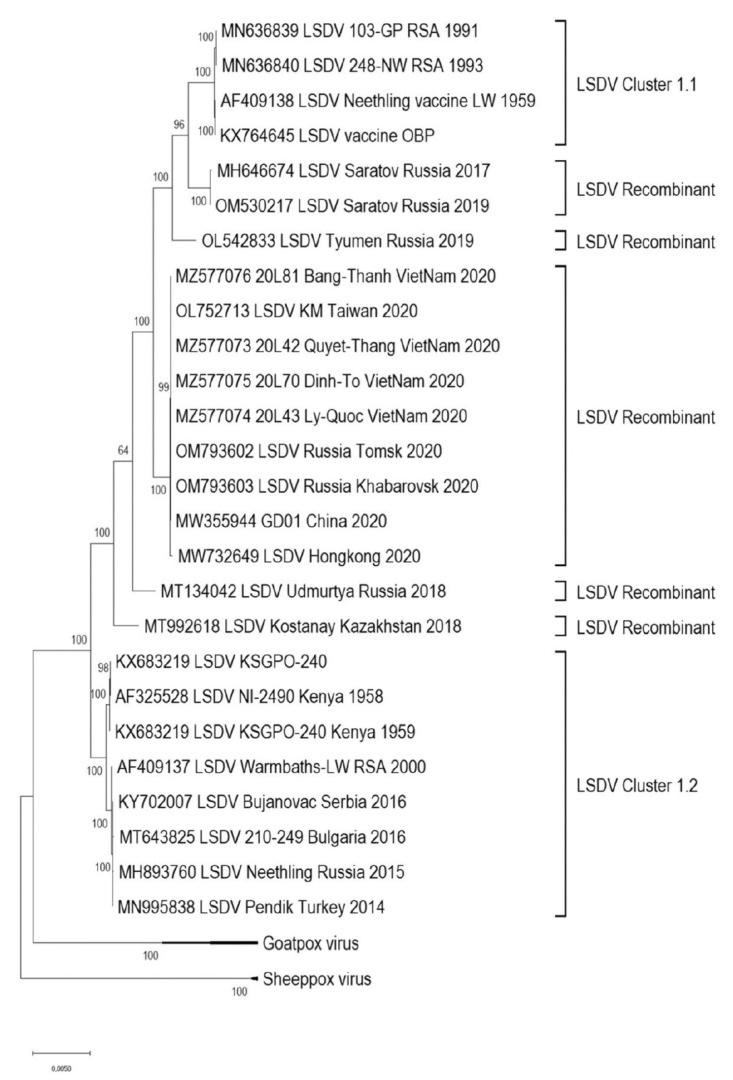
Maximum likelihood phylogenetic tree representing genetic relationship of the complete genome sequences of 26 LSDVs, 2 GPVs and 2 SPPVs with permission from A. Sprygin [78].

In the past, LSDV genomes were reported to be relatively stable. For example, when comparing a virulent LSDV field-strain from South Africa and a virulent Kenyan 2490 strain, there were only 38 amino acid differences in 29 of the 156 putative genes and mostly 1 to 3 amino acid changes in the variable terminal regions. There were also six deletions and eight insertions detected [66]. In a study comparing the prototype, the Neethling-WC/RSA/1957 strain from which the vaccine was derived, and the vaccine prepared in 1988 (LW-1959), only seven single-nucleotide polymorphisms were identified. In addition, the oldest available LSDV isolate LSDV/Haden/RSA/1954 was sequenced and compared with Neethling-type commercial vaccine viruses and had eight SNP differences. Both viruses formed part of Cluster 1.1 (refer to Figure 4). It is estimated that the substitution rate of LSDV is in the range 7.4 × 10^−6^ substitutions/site/year. Natural genetic drift is responsible for the differences between the phylogenetic clusters. In the natural population, there is a high frequency of synonymous mutations whereas highly cell passaged viruses have an increase in non-synonymous mutations. LSDV has high genetic stability and is well adapted to the cattle host [67]. This differs from the recent human outbreak of monkeypox virus, which is still adapting to the new human host, where point mutations continue to accumulate at an unexpected rate [68].

However, it is also well accepted that poxviruses can recombine under specific conditions to give chimeric viruses. The mechanisms include recombination, and horizontal gene transfer to acquire new genes [69]. The first experiments to show recombination between different orthopoxviruses were conducted in the 1960s [70,71,72]. Marker rescue studies were carried out to demonstrate how homologous recombination could be used to generate recombinant poxviruses with novel inserts [73] and this subsequently evolved into making recombinant poxvirus-based vaccines [74]. Poxviruses under specific stresses can duplicate genes to adapt to new conditions [69,75,76]. However, there was minimal evidence that Capripoxviruses would recombine in the field [77]. Prior to 2017, the LSDVs could be grouped into two clusters that were separate from GPPV and SPPV [78] (refer to Figure 4).

With this background, it was unexpected to identify recombinant LSDV in the field. Kazakhstan used a vaccine called Lumpivax (KEVEVAPI, Narobi, Kenya) to control an LSD outbreak (2017–2019) and following this, there was an emergence of vaccine-like LSDV recombinants in Kazakhstan and neighbouring China and Russia. One of the first reports of virulent vaccine-like strains of LSDV was from a LSD outbreak in Russia in 2017. An in-depth analysis of one of the recombinants, LSDV RUSSIA/Saratov/2017 identified 27 recombination events and included fragments from wild-type field strains of Capripoxviruses [79]. It was speculated that the vaccine being used in neighbouring Kazakhstan had reverted to virulence since, at that time, attenuated LSDV-based vaccines were not permitted in Russia [80]. It was further hypothesised that the recombinant LSDV had a fitness advantage in the colder weather conditions of the northern latitudes [78]. 

**Figure 5:**
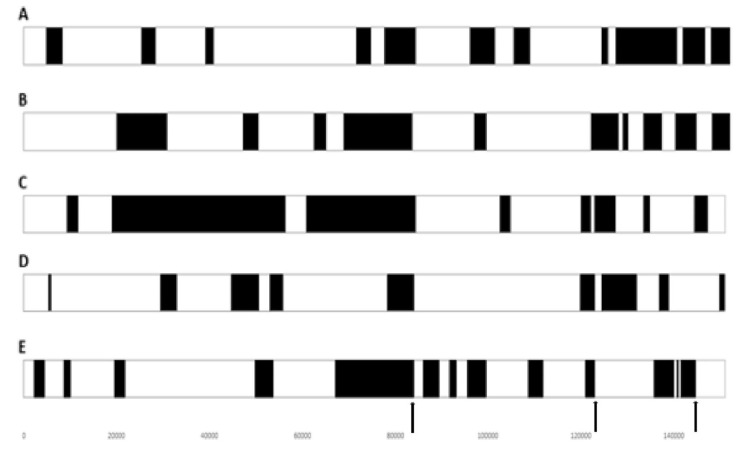
Graphical representation of the position of the predicted recombination events across the five strains with the highest statistical significance. The events are indicated in black in the white backbone of the viral genome. Possible regions selected for by recombination are indicated by arrows (positions 80,000 bp, 124,000 bp and 141,000 bp). (**A**) LSDV/KZ-Konstanay/Kazakhstan/2018. (**B**) LSDV/Russia/Tyumen/2019. (**C**) LSDV/Russia/Udmurtya/2019. (**D**) LSDV/Russia/Saratov/2017. (**E**) LSDV/GD01/China/2020 with permission from A. Sprygin [78].

Further analysis of emerging recombinants showed a number of unique recombinants that emerged before 2020. From 2020 onwards, all circulating strains in Russia and South-Eastern Asia belonged to a single lineage radiating out in the region. After 2017, with the emergence of recombinant LSDVs from the Russian Federation, Kazakhstan, China and Vietnam, further distinct clusters were seen (refer to Figure 4) [78]. The recombination events were predicted to be between parental strains, a Kenyan KSGPO-like and LSDV/Vaccine/LW-1959-like strain. A comparison of recombination sites in five recombinant viruses demonstrated a unique mosaic pattern for each. However, there were some common break points at positions 80,000 bp, 84,000 bp, 124,000 bp and 141,000 bp (refer to Figure 5) [78]. These unique recombinant viruses illustrate the importance of whole genome sequencing to track poxviruses and conduct molecular epidemiology. 

Further proof on the origin of the recombinant LSDVs came when the analysis of two batches of the vaccine Lumpivax found it to be a combination of different Capripoxviruses including a Neethling-like LSDV vaccine strain, a KSGP-like LSDV vaccine strain and a Sudan-like GTPV strain [27]. In this study, they directly analysed the genomes present in the vaccines and found that the vaccine-like recombinant strains could be divided into four groups. They each arose from multiple recombination events with a large number of genetic exchanges ranging between 126 and 146. Since the viruses were sequenced directly from the vaccine lots, it is likely the recombination occurred during the manufacturing process. Not all the recombinants would be expected to have equal fitness in animals and the most fit would be selected and transmitted. The emergence of recombinant vaccine-like LSDV strains in Asia is now thought to originate from spill-over from animals vaccinated with Lumpivax, highlighting the importance of vaccine quality control [27]. Whole genome sequencing of vaccine stocks should be part of the quality checks.

### 1.8. Next Generation Vaccines Being Developed for LSD including Modified LSDV, Inactivated LSDV and Subunit LSD Vaccines as well as mRNA Based Vaccines

When LSD first emerged in India, there was no commercial LSD vaccine and so the GTPV vaccine was used. Recently, a live-attenuated LSD vaccine, named Lumpi-ProVacInd, has been developed from a circulating LSDV isolate closely related to the Kenyan-type LSDV strain. The virus was attenuated by passaging fifty times in Vero cells. The vaccine protects from LSDV challenge and large clinical trials have taken place indicating that the vaccine is safe in cattle, buffalo and lactating pregnant animals. The vaccine is now being commercialised for use in Asia [81]. 

Targeted approaches to knock-out virulence genes have been used to create an attenuated LSDV for vaccine use. Kara et al. (2018) deleted two genes from a virulent LSDV in separate experiments to attempt to attenuate LSDV. These genes coded for homologues of an interleukin 10-like (LSDV005) and interferon- γ receptor-like genes (LSDV008), respectively. The resulting knockout LSDVs were still virulent and there were severe post-vaccinal reactions and febrile responses were observed for both constructs, with the interferon- γ receptor-like gene knock-out LSDV being more virulent than the interleukin 10-like knockout LSDV [82]. Chervyakova et al. (2022) knocked out four LSDV genes to attenuate the virulent LSDV Atyrau/KZ. These genes included the LSDV005 gene which encodes an interleukin-10-like protein; LSDV008, a homologue of the vaccinia virus B8R gene which encodes an interferon-γ receptor-like protein; LSDV142, a homologue of the N1L gene of the vaccinia virus, which encodes a protein of the Bcl-2-like protein family and inhibits both apoptosis and activation of the pro-inflammatory transcription factor NF-κB (nuclear factor kappa B); and LSDV066, a gene which encodes thymidine kinase. Bovine interleukin-18 mRNA was inserted into the thymidine kinase locus to knock this gene out, whereas the other three genes were deleted. The resulting candidate vaccine, LSDV Atyrau-5BJN (IL18), was safe and protected from virulent LSDV challenge [83]. In our laboratory, the superoxide dismutase gene (SOD) of the nLSDV vaccine was replaced with a synthetic, stabilised SOD gene to generate an nLSDV that potentially has further reduced virulence, as well as improved immunogenicity [84].

There is some hesitancy to introduce live attenuated LSDV vaccines into areas with no LSDV, not only for safety considerations, but also because of the impact on the “disease free status” of the country [85]. Therefore, there has been an effort to develop inactivated and subunit vaccines [86,87]. As a result, developments have been made to produce safer LSDV vaccines and to further improve their immunogenicity. Chemically inactivated nLSDV vaccines with adjuvants have been developed as safer alternatives to live nLSDV [85,86,88,89,90]. The combination of a low-molecular-weight copolymer adjuvant and inactivated LSDV, at titres similar to live vaccines, has given protection from LSDV challenge [85].

The state of New South Wales of Australia is working with the biotech company Tiba Biotech to develop LSDV mRNA-based vaccines [91]. This project is in the early stages of development. Since Australia is presently free of LSD, strategies based on other approaches than the live attenuated LSDV vaccine are desirable. 

### 1.9. LSDV as a Vaccine Vector

Modern candidate recombinant vaccines are based on DNA or mRNA, subunit proteins, attenuated pathogens, inactivated pathogens or virus-vectored platforms. Live virus vectors encoding foreign antigens have many advantages compared to the other strategies: they can induce both humoral and cellular responses, as opposed to subunit protein-based vaccines that are often limited to eliciting antibody responses; by nature, they are highly immunogenic and therefore usually do not require adjuvants; cellular uptake is generally more effective due to active infection, compared to DNA vaccines that need to translocate to the host nucleus; and some may overcome logistical issues as they can be freeze-dried [91,92,93]. Poxviruses, particularly safe derivatives of vaccinia virus (VACV), have been used extensively as live vaccine vectors. Their genomes can tolerate very large insertions, enabling one virus to encode multiple antigens; VACV can hold the entire 27.3 kb genome of a human coronavirus [94]. Poxviruses complete their replication in the host cell’s cytoplasm, therefore there is less concern of host genomic integration of foreign genes [4].

Various methods exist to construct recombinant poxviruses. Modern, less frequently used methods include the generation of synthetic viruses by ligation of synthesised DNA fragments or the use of CRISPR/Cas9 [95,96]. Poxvirus genomes are not repaired well following Cas9 cleavage and so the use as an editing tool is not efficient. However, it works well as a selection agent to select recombinants and by speeding up isolation of recombinant poxviruses [97].

**Figure 6:**
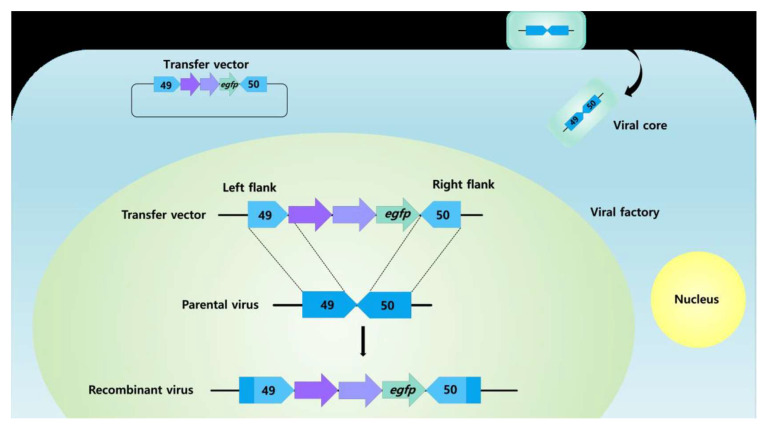
Schematic diagram of the generation of a recombinant poxvirus by homologous recombination of parental virus and a transfer vector. The plasmid encoding the transfer vector contains two antigen genes (purple arrows), an enhanced green fluorescent protein gene (egfp) as a marker gene (green arrow) as well as left and right flanks (49 and 50 flanks, respectively; blue arrows). Overall, 49, left flank of the transfer vector, ±300 bp of the 3′ end of 049 ORF of the LSDV genome; 50, right flank of the transfer vector, ±300 bp of the 3′ end of 050 ORF of the LSDV genome. All genes expressed by poxviruses are under the control of poxvirus promoters [99] (From student thesis where I am the superviso).

Traditionally, however, recombinant poxviruses are made via homologous recombination between a DNA plasmid encoding the foreign vaccine antigen(s) together with poxvirus promoters, and the parent poxvirus genome [98] (Figure 6).

An insertion site is chosen for the introduction of the foreign genes. The site should not be a mutational ‘hot-spot’ as this may cause the foreign genes to mutate or be deleted, and integration of the foreign genes should not disrupt sequences essential to LSDV. The LSDV thymidine kinase and ribonucleotide reductase genes have been used previously by the insertion of foreign sequences into these sites, resulting in disruption of these genes [100,101,102]. These sites are thought to further attenuate the vaccine and result in slower growth and so other insertion sites are more desirable. A putative non-essential gene encoding a G-protein-coupled chemokine receptor subfamily homologue has been tested as a site for homologous recombination [103]. The region between the convergent LSDV open reading frames (ORFs) 49 and 50 have also been used without gene disruption [20,104,105]. 

Inserted foreign genes require poxvirus promoters for the recognition of the poxvirus transcriptional machinery. Various promoters from other poxviruses have been shown to function in LSDV (Table 1) [24,106]. The timing of expression is also regulated by the promoter, such as whether it is expressed during the early stage of the poxvirus lifecycle, the late stage, or both. Choice of promoter may also affect the immunogenicity of the controlled antigen; for example, promoters that give early expression may enhance T-cell responses to the expressed antigen [107]. In addition, the stop codons of genes need to be followed by poxvirus transcription terminator sequences TTTTTAT, TTTTTCT or TTTTTGT [108]. Excluding the terminator can reduce transcription of the gene.

**Table 1:** Summary of various poxvirus promoters that have been shown to be functional in LSDV.

Promoter	Origin	Type	Sequence (5′ to 3′)	References
mH5	VACV, modified	Early-late	AAAAATTGAAAATAAATACAAAGGTTCTTGAGGGTTGTGTTAAATTGAAAGCGAGAAATAATCATAAATAA	[109]
pLEO	VACV, modified	Early-late	TTTTATTTTTTTTTTTTGGAATATAAATATCCGGTAAAATTGAAAAAATATACACTAATTAGCGTCTCGTTTCAGACGCTAG	[110]
p7.5	VACV, modified	Early-late	CCAAACCCACCCGCTTTTTATAGTAAGTTTTTCACCCATAAATAATAAATACAATAATTAATTTCTCGTAAAAGTAGAAAATATATTCTAATTTATTGCACGG	[111]
p11	VACV	Late	TTTCATTTTGTTTTTTTCTATGCTATAA	[112]
pSS	Synthetic	Early-late	AAAATTGAAATTTTATTTTTTTTTTTTGGAATATAAATA	[113]
mFP	Fowlpox, modified	Early-late	AGAAAAATATCCTAAAATTGAATTGTAATTATCGATAATAA	[20]

Reporter genes such as *E. coli* β-galactosidase (LacZ) or fluorescent markers such as GFP can be included to visualise cells infected with the recombinant LSDV [101,105]. Selection markers can also be used to enable the recombinant to grow in cell lines or conditions that the parent virus cannot. Mycophenolic acid tolerance induced by expression of the *gpt* gene of *E. coli* (ECOGPT) has been used in rLSDV-infected cells as a method of selection [9]. This method enriched for recombinants but did not exclude all parent virus. The VACV K1L gene, which rescues modified vaccinia Ankara (MVA) growth in rabbit kidney 13 (RK-13) cells, can also rescue LSDV growth in RK-13 cells, although growth tends to be slow [24,114]. 

LSDV-vectored cattle vaccines would be a major advantage, as these would provide dual or multivalent vaccines [115]. There are numerous recombinant Capripoxvirus vaccines that have been constructed (Table 2) and tested for immunogenicity as well as, in some cases, in challenge experiments. KS-1 recombinants have been successful in protecting against rinderpest virus [116], rift valley fever virus [117], Peste-des-petits-ruminants virus [118] and have provided partial protection from blue tongue virus challenge [119]. These experiments were performed in sheep and in many cases the recombinant KS vaccines also protected from SPPV challenge. KS-1 was originally thought to be of SPPV origin, but sequencing revealed that it was actually an LSDV derived vaccine which retained some virulence in cattle [120]. LSDV expressing rabies glycoprotein protects from rabies virus challenge in rodent models [6,121], as well as inducing protective levels of rabies virus antibodies in cattle [101]. LSDV expressing bovine ephemeral fever virus (BEFV) proteins induced good BEFV and LSDV neutralising antibody responses in rabbits and cattle. The cattle were protected from LSDV challenge [104]. Despite the success of recombinant LSDV vaccines, there are still none that have been commercialized. This is probably due to the poor profit margins in the affected communities where the diseases are prevalent; however, this may change with the expansion of LSD across Asia. 

**Table 2:** Experimental vaccines using Capripoxvirus as a live virus vector.

Target Pathogen	Antigen Expressed	Capripoxvirus Backbone	Reference
Rindepest Virus	Rindepest Virus H and F gene	KS-1 *	[122]
Peste-des-petits-ruminants virus (PPRV)	hemagglutinin (H) or the fusion (F) protein gene of PPRV	KS-1 *	[118]
Blue Tongue Virus (BTV)	BTV VP7	KS-1 *	[119]
Rift Valley Fever Virus (RVFV)	RVFV Gn and Gc glycoproteins	KS-1 *	[117]
HIV	Grttn polyprotein	nLSDV	[106,123]
	Env and Gag	nLSDV with SODis	[105]
Rabies virus (RABV)	RABV Glycoprotein	nLSDV	[6]
Mokola virus (MOKV), West Caucasian bat virus (WCBV), Rabies Virus (RABV)	RABV, MOKV and WCBV glycoprotein genes	nLSDV	[121]
Rift valley fever virus (RVFV)	RVFV Gn and Gc glycoproteins	nLSDV	[102,124,125]
Bovine ephemeral fever virus (BEFV)	BEFV Glycoprotein	nLSDV	[102,124,125]
	Glycoprotein	nLSDV with either SODis or ΔSOD	[104]
	Glycoprotein and Matrix		

nLSDV: Neethling strain of LSDV; SODis: stabilised superoxide dismutase gene; ΔSOD: deleted superoxide dismutase gene. * KS-1 was originally thought to be of SPPV origin, but sequencing revealed that it was actually an LSDV derived vaccine [120].

LSDV is a host restricted virus and does not complete the replication cycle in non-ruminant hosts. LSDV expressing HIV proteins has been shown to be immunogenic in rhesus macaques and boost the responses primed by a MVA vectored vaccine. The combination regimen induced high-magnitude, broad and balanced CD4(+) and CD8(+) T-cell responses, and transient activation of the immune response. These studies support further development of LSDV as a vaccine vector [123] in non-ruminant hosts. LSDV expressing HIV Gag and Env and tested in combination with MVA induced good antibody responses in rabbits [105]. LSDV was injected into mice and the response after 24 h was compared with that of five host-restricted poxvirus species from three genera, namely Canarypox virus (CNPV), Fowlpox virus (FWPV), MVA and two novel South African avipoxviruses. These six viruses produced qualitatively and quantitatively distinct host responses with LSDV, followed by MVA, inducing the greatest interferon (IFN) response [126]. This illustrates that poxvirus vectors differ and may have different strengths as vaccine vectors. 

## 2. Conclusions

Lumpy skin disease, an important notifiable disease of cattle, was originally an African disease and is now becoming established in large parts of the Middle East and Asia. There are available vaccines and the most effective are those based on attenuated LSDV. Vaccination remains the best way to control outbreaks. Given the spread of LSDV by biting insects, it is important to vaccinate to protect against LSD as well as control the insect population around infected animals. Next generation LSD vaccines are being developed which include further attenuation of LSDV and mRNA-based vaccines. In addition, LSDV is being investigated as a vaccine vector with the possibility of multivalent vaccines being developed.

## 3. Patents

The authors have filed patent applications on various nLSDV-based vaccines: CT/IB2019/054090, PA166012PCT; PCT/IB2022/056970, PA175637/PCT; PA179963/P, PA179694/P, PA176615/P.

## Data Availability

Not applicable.
